# Influence of social media use on life satisfaction among deaf and hard of hearing college students: a mediating role of self-esteem and perceived social support

**DOI:** 10.3389/fpsyg.2025.1554621

**Published:** 2025-03-03

**Authors:** Xinyi Huang, Liang He

**Affiliations:** School of International Studies, Hangzhou Normal University, Hangzhou, China

**Keywords:** life satisfaction, deaf or hard of hearing, perceived social support, self-esteem, college students

## Abstract

**Introduction:**

As social media continues to gain popularity and living standards improve, people are becoming increasingly concerned with their quality of life, highlighting the importance and urgency of exploring the relationship between social media and well-being. At the meantime, the gap between majority and minority groups is widening in digital era. However, there is currently insufficient research on the use of social media by D/HH (Deaf or hard of hearing) individuals and its impacts. There is also a lack of international attention on China, which has the largest population of D/HH individuals.

**Methods:**

The main objective of this research was to explore how social media use impacts D/HH college students’ well-beings, and this study recruited 320 Chinese hearing-impaired social media users and conducted a questionnaire survey using the SWLS (Satisfaction with Life Scale), MSPSS (Multidimensional Scale of Perceived Social Support), and RSES (Rosenberg Self-Esteem Scale) scales. The study delved into the reasons for social media use among this population, the relationship between social media use frequency and life satisfaction, and the potential mediating roles of self-esteem and perceived social support.

**Results:**

The findings revealed that the primary reasons for social media use among hearing-impaired individuals include communication, acquiring information and knowledge, and relaxation. Moreover, there is a positive correlation between social media use frequency and life satisfaction, perceived social support, and self-esteem, with both direct and indirect effects. These results align with earlier studies and our comprehension of how social media use boosts life satisfaction, potentially advancing research in this field.

**Discussion:**

This paper explores in depth media accessibility, the mediating mechanisms of perceived social support and self-esteem, and their impacts on life satisfaction and other mental health issues of D/HH.

## Introduction

The rapid advancement of internet technologies, including big data, cloud computing, and artificial intelligence, has significantly transformed social interactions and everyday life. These innovations have facilitated the widespread integration of digital tools, leading to the pervasive presence of artificial intelligence in various aspects of society. As a result, social media has become an essential platform for communication, fundamentally reshaping interpersonal relationships. According to Statista (2024), as of October 2024, there were 5.22 billion social media users worldwide, accounting for 63.8% of the global population. Simultaneously, the number of hearing-impaired individuals worldwide is increasing. The World Health Organization’s World Report on Hearing (2021) highlights that over 1.5 billion people globally experience some degree of hearing loss, representing approximately 5% of the world’s population. This figure is projected to rise, with over 700 million people—one in ten—expected to have disabling hearing loss by 2050.

Against this backdrop, the studies about social media and its impacts on users’ perceived social support, self-esteem, and life satisfaction have emerged as a significant area of scholarly inquiry (Hawi and Samaha, 2017; Jan et al., 2017; Hwang and Kim, 2015; Çivitci and Çivitci, 2009). Studies have focused on various aspects of social media use, including frequency, intensity, and the number of SNS (Social Network Sites) friends (Oshio et al., 2020; Kaplan and Haenlein, 2010), and highlighted its vital role in providing social connection among global users (Kietzmann et al., 2011).

However, many of the existing studies mentioned above predominantly concentrate on unimpeded majority populations or young adults and undergraduates, often employing a narrow focus on specific platforms, such as Facebook, neglecting the experiences of individuals with disabilities-a population that particularly needs to snatch help from social media and has the potential to foster social connection. Addressing the limitations of previous studies that lack data on Chinese hearing-impaired individuals and only use a single social media platform, our research focuses on China, which has the largest population of hearing-impaired individuals (Data from China’s Second National Disability Survey Leadership Group, Second National Disability Survey Office, 2024 reveals that China has the largest population of individuals with hearing disabilities globally, reaching a staggering 27.8 million), and includes the use of various social media to examine the relationship between social media use frequency and life satisfaction among D/HH college students, considering the mediating effects of perceived social support and self-esteem.

The current study aims to investigate the relationship between the frequency of social media use and life satisfaction among D/HH (Deaf and Hard-of-Hearing) college students, taking into account the mediating roles of perceived social support and self-esteem.

## Literature review

### Reasons for social media use

The motivations behind social media use are multifaceted, yet users will seek for the fulfillment of their specific needs and gratifications (Lariscy et al., 2011). Individuals leverage social media platforms to search for and access diverse information; furthermore, these platforms provide a convenient channel for establishing extensive or intimate social connections with others around the globe (Kietzmann et al., 2011). Through activities such as browsing short videos, watching live streams, and participating in online games, users find respite from demanding schedules, achieving relaxation and enjoyment.

Based on broad observation and research, Whiting and Williams (2013) listed eight key reasons for media consumption; for D/HH groups, the need to establish social connections is particularly pronounced. The virtual space offers a greater degree of accessibility in communication because of the visual images which can be seen instead of listening, mitigating the challenges posed by hearing loss. Crucially, social media provides a relatively equitable and respectful environment where privacy is better protected, allowing them to forge genuine and enduring interpersonal relationships without necessarily disclosing their disability which they are afraid of exposing during voice call or offline communication (Eichengreen et al., 2016; Hunsinger and Senft, 2013). Specifically, D/HH individuals utilize social media as a convenient channel to receive health information (Kushalnagar and Kushalnagar, 2018); and they establish targeted online communities so that they can get in touch with their companions who are suffering from the same hardship. Therefore, the current study proposes the first research question: RQ1: What are the major reasons for social media use among Chinese D/HH college students?

### Relationships between social media use and life satisfaction

With the unprecedented advancement of social productivity and the progressive evolution of human consciousness in the digital age, individuals are increasingly introspective about the alignment between their lived experiences and internal aspirations. This heightened self-awareness extends beyond mere material satisfaction to encompass a more holistic evaluation of life quality. Societal factors is of growing significance, especially the pervasive influence of emerging technologies on the disabled’s increasing participation in virtual spaces (Hunsinger and Senft, 2013), and their subjective quality of life (Hintermair, 2008). In the contemporary era dominated by social media and digital connectivity, studies about SNS use and its impacts on life satisfaction, perceived social support, self-esteem and community connection has emerged as a critical area of scholarly inquiry (Paglieri et al., 2023; Alessandra Gaia et al., 2020; Escobar-Viera et al., 2020; Miller, 2017). This intersection of digital technology and subjective well-being presents a complex paradigm that merits rigorous academic investigation, particularly given the unprecedented degree of technology adoption and integration into daily life routines.

Life satisfaction, conceptualized as a conscious evaluative process through which individuals assess their quality of life based on self-selected criteria (Pavot and Diener, 1993, 2008), represents a fundamental construct in well-being research. While the parameters for assessing life satisfaction are inherently heterogeneous and individually determined, the need for methodological standardization led Diener et al. (1985) to develop the Satisfaction with Life Scale (SWLS). The scale’s versatility and robust psychometric properties have enabled its application across diverse populations, as exemplified by Hintermair (2008) and Wilson et al.’s (2020) investigation of life satisfaction among deaf individuals. At present, people tend to communicate via social media to establish social connection which is regarded as an important aspect of enhancing the level of life satisfaction (Antonucci et al., 2017; Hutto et al., 2015). However, empirical researches examine the nexus between social media use and life satisfaction continues to present contradictory results. While one stream of research suggests that social media use plays a positive role on life satisfaction (Oshio et al., 2020; Alessandra Gaia et al., 2020), with documented cascading effects on individual’s psychosocial well-being, a competing body of scholarship has cast doubt on the beneficial impacts of social media use on life satisfaction (Oh et al., 2014; Valenzuela et al., 2009). As D/HH catching increasing attention, scholars turn their views on D/HH groups’ well-being and life satisfaction (Glickman, 1993). Initially, Glickman and Carey (1993), based on Helms’s (1990) Racial Identity Development Theory, proposed the DIDS (Deaf Identity Development Scale). This scale suggests that deaf individuals undergo identity development through four stages: initially internalizing negative hearing perspectives and viewing deafness as a medical pathology, experiencing marginalization (confusion about belonging), immersion (uncritically accepting all “deaf” identities and denigrating those with normal hearing/values), and finally reaching an integrated (bicultural) stage. However, the scale has difficulties distinguishing subtle changes in individual attitudes and tends to conflate different subscale identities. Therefore, Maxwell-McCaw and Zea (2011) developed the Deaf Acculturation Scale (DAS) to address these deficiencies, creating a more reliable model for assessing deaf cultural adaptation. Some results indicate that individuals with hearing impairments are more likely to lack a sense of belonging due to exclusion from hearing environments, which impacts their mental health and cognitive functioning. Social media has altered this situation. Paglieri et al. (2023) focused on the special social media use circumstances of elderly deaf individuals, proving there is positive relationship between social media use and life satisfaction; Lambez et al. (2020) discussed the relationship between cultural adaptation models and emotional disorders in D/HH and hearing populations; other studies discussed the advantages and disadvantages of CI for social connections among D/HH individuals (Bradfield, 2021; Sparrow, 2005). Considering the previous results, this study has the following hypothesis:

Hypothesis 1: The frequency of social media use is positively correlated with life satisfaction among Chinese D/HH college students.

### The mediating effect of perceived social support and self-esteem

While examining the relationship between SNS use and life satisfaction, Oshio et al. focused on the mediating effect of perceived social support and suggested that social media use can enhance subjective well-being through its positive impact on perceived social support (2020). Social support is well-known as the “various resources provided by one’s interpersonal ties” (Cohen and Hoberman, 1983, p. 100), including support from family, friends and significant others, which is essential to individual’s quality of life, because of its alleviation potential while enduring emotional and physical situation (Michel et al., 2010). Besides, one of the motivations people use social media is to obtain social support which can be divided into structural (i.e., size and shape of interpersonal connections) and functional (i.e., perceived availability of interpersonal resources) (Sherbourne and Stewart, 1991). Given that perceived social support offers better psychological adjustment and relevant and valid representation (Li et al., 2015; Trepte et al., 2015), the current study examine perceived social support’s mediating effect. Social media use and perceived social support are positively correlated, according to both theoretical and empirical research. Theoretically, active interpersonal contact is a key tactic for preserving connections (Coleman, 1988; Crocker and Canevello, 2008), and social media use provides a convenient platform for such interactions. Empirically, a positive association between social media use and perceived social support has been demonstrated by a number of studies utilizing survey data (Paglieri et al., 2023; Lin et al., 2022; Lee and Cho, 2019).

Self-esteem refers to “an individual’s positive or negative evaluation of himself or herself” (Smith et al., 2014, p. 107). Researchers have long considered self-esteem to be a crucial measure of mental health and quality of life (Hintermair, 2008), in order to assess it visually, Rosenberg developed the Rosenbery Self-Esteem Scale (RSES) in 1956. The preponderance of research investigating the connection between self-esteem and social media utilization has demonstrated that individuals with lower self-esteem are inclined to engage more frequently with social media platforms in an attempt to bolster their self-image and elevate their self-esteem (Blachnio et al., 2016; Denti et al., 2012). Correspondingly, previous research indicates social media use gives users more chances to gain positive feedback, relieve pressure and enhance high-level of self-esteem (Yang et al., 2021); it comes as no surprise self-esteem has a positive correlation with life satisfaction (Hawi and Samaha, 2017). In addition, sociometer theory posits that self-esteem functions as a gauge of interpersonal relationships (Leary et al., 1995; Kirkpatrick and Ellis, 2001) and it is perceived as an internal reflection of social support. Thus, self-esteem might be influenced by perceived social support at first and then impacts life satisfaction. As for relevant research about D/HH’s using situation, the amount is not sufficient, Schäfer and Miles suggests that the more D/HH participants use social media, the higher their self-esteem levels (2023).

In shorts, historical research suggests that perceived social support and self-esteem serve as mediating variables in the relationship between social media use and life satisfaction. Additionally, self-esteem may function as a mediator between social support and life satisfaction. Hence, this study proposed the following hypotheses:

Hypothesis 2: The relationship between social media use frequency and life satisfaction is mediated by perceived social support and self-esteem among Chinese D/HH college students.

Hypothesis 3: The mediators have sequential effect, which refers to social media use frequency has positive impact on social support, and social support is correlated with self-esteem, which in turn enhances life satisfaction among Chinese D/HH college students.

### Overall review of previous researches on D/HH

Previous research has predominantly focused on young individuals or those without physical barriers to communication. However, for the D/HH (Deaf and Hard of Hearing) population, the opportunities provided by social media for information acquisition and social connection are extraordinarily invaluable. Social media significantly mitigates the discomfort and inferiority they feel in offline face-to-face interactions.

Overall, research on social media use and its effects has been progressing, but it also has certain limitations. Although studies have begun to address the D/HH population, they remain relatively fragmented and superficial, lacking detailed analysis of how different variables interact and not emphasizing mediating effects. More importantly, as the largest developing country and the nation with the largest number of hearing-impaired individuals, research on the social media usage and life satisfaction of hearing-impaired individuals in China is crucial. Previous studies have primarily focused on social media use and its relationship with other variables among users in Europe, the United States, or other developed countries, neglecting the situation of users in developing countries. This represents a significant gap in the academic field that needs to be addressed.

## Method

The study of social media use among D/HH individuals in China is currently an underrepresented topic in research. This study utilizes a questionnaire survey method to collect and analyze quantitative data. The quantitative data obtained from survey responses will help understand the various ways and reasons that Chinese D/HH college students use social media and how the condition may affect them. These insights are valuable.

### Participants and recruitment

Participants include three hundred and twenty citizens recruited from Hangzhou, Zhejiang Province. Participants aged from 17 to 29 (M = 21.29). The questionnaire was designed basing on established scales and published on Wenjuanxing (an online survey platform), and then distributed by the teachers of D/HH college students to facilitate the completion of the survey. One hundred and eighty-three of them were females, and all the participants were social media users. Due to the uneven development of internet access between urban and rural areas in China, people living in rural areas may have fewer opportunities to access the internet. Therefore, a simple investigation of this factor will also be conducted here.

### Measures

Since that there is no further substantial development among the following measures, we continue to use the original versions.

#### Social media use frequency

The Active Social Networking Sites Usage Questionnaire (ASUQ, Ding et al., 2017) was employed to evaluate the frequency of SNS use. Participants rated their usage on a 5-point Likert scale, with options ranging from 1 = “Never” to 5 = “Always.” The overall score is the mean of all item scores, with higher scores signifying a greater frequency of social media use.

#### Perceived social support

The present study utilizes the Multidimensional Scale of Perceived Social Support (MSPSS; Zimet et al., 1988) to gauge perceived social support. The MSPSS is a 12-item self-report instrument developed by Zimet to evaluate perceived support from significant others (*α* = 0.91), family members (α = 0.87), and friends (α = 0.85), which demonstrated high reliability of this scale. Respondents rated their agreement on a 7-point Likert scale, ranging from 1 = “strongly disagree” to 7 = “strongly agree.” Example items include: “There is a special person who is around when I need,” “I have friends with whom I can share my joys and sorrows,” as well as “I get the emotional help and support I need from my family.” The total score is the sum of all item scores, with higher scores indicating a greater level of perceived social support. Additionally, a previous study by Paglieri et al. (2023) employed this measure and confirmed its high validity and reliability (*α* = 0.88, re-test α = 0.85).

#### Self-esteem

The Rosenberg Self-Esteem Scale, developed by Rosenberg (1965), is utilized to measure self-esteem. Participants indicate their level of agreement on a Likert scale ranging from 1 = “strongly disagree” to 4 = “strongly agree.” The RSES comprises ten items, with positive statements (items 1, 3, 4, 7, and 10) and negative statements (items 2, 5, 6, 8, and 9) alternating to provide a holistic evaluation of an individual’s self-perception and personality. The aggregate score is the sum of all item scores, with higher scores reflecting greater levels of self-esteem. Schäfer and Miles (2023) made minor adaptations to this scale for use with D/HH participants, discovering that an increased number of social media accounts correlated with higher self-esteem among the D/HH participants. For the current sample, the Cronbach’s coefficient *α* of RSES was 0.95.

#### Life satisfaction

Shin and Johnson (1978) define life satisfaction as “a global assessment of a person’s quality of life according to his chosen criteria”. Diener et al. (1985) developed Satisfaction with Life Scale, an instrument designed to quantitatively assess individual’s perception of their life satisfaction. This scale proposed five statements: “In most ways my life is close to my ideal,” “The conditions of my life are excellent,” “I am satisfied with my life,” “So far I have gotten the important things I want in life,” “If I could live my life over, I would change almost nothing.” to evaluate their feelings about these statements, participants need to mark their level of agreement on a 7-point Likert scale. The 7-point scale is: 1 = strongly disagree, 2 = disagree, 3 = slightly disagree, 4 = neither agree nor disagree, 5 = slightly agree, 6 = agree, 7 = strongly agree. For the current sample, the Cronbach’s coefficient α of life satisfaction was 0.94.

### Procedures

Participants were recruited using a convenience sampling method. A total of 320 D/HH college students voluntarily participated in this study without compensation. The survey was administered in a home environment and required approximately 20 min to complete. Prior to completing the main scales, respondents addressed several demographic questions. All participants completed the survey anonymously. The instructions clarified that the data would be used exclusively for research purposes, and participants retained the right to withdraw at any point if they felt uncomfortable with any of the questions.

The survey content included basic demographic information, the amount and frequency of social media usage, reasons for usage (Whiting and Williams, 2013), seeking offline activities, social support, self-esteem, and life satisfaction (Diener et al., 1985; Wilson et al., 2020). Each measurement scale was carefully selected to obtain the data necessary to answer the research questions of this study.

### Data analysis

This study utilized IBM SPSS Statistic 22.0 software for statistical analysis, covering various methods including frequency statistics, difference analysis, descriptive statistics, correlation analysis, multiple linear regression, and mediation effect analysis. Frequency statistics were used to analyze the distribution of basic characteristics such as age, gender, household registration, and disability status. Independent samples *t*-tests were conducted for difference analysis to explore gender differences in social media use frequency, perceived social support, self-esteem, life satisfaction. Descriptive statistics further detailed the respondents’ basic information. Pearson correlation analysis revealed the relationships among self-esteem, life satisfaction, perceived social support, and social media use frequency. Multiple linear regression analysis established a predictive model of social software usage frequency on life satisfaction. Mediation effect analysis, based on the Bootstrap method, examined the mechanisms of how the frequency of social media use impacts life satisfaction (see Table 1).

**Table 1:** Sociodemographic characteristics of participants at baseline.

Characteristics	Value	Number	Percentage
Age	17	1	0.3%
	18	4	1.2%
	19	36	11.2%
	20	63	19.6%
	21	89	27.8%
	22	67	20.9%
	23	31	9.6%
	24	16	5.0%
	25	5	1.2%
	26	4	0.3%
	27	2	0.6%
	29	2	0.6%
Gender	Male	137	42.8%
	Female	183	57.1%
Type of registered permanent residence	Rural household registration, live in the countryside	198	61.8%
	Urban residence certificate, live in cities	46	14.3%
	Rural household registration, live in cities	76	23.7%
Disability status^a^	Hearing Disability Class I	234	73.1%
	Hearing Disability Class II	70	21.8%
	Hearing Disability Class III	10	3.1%
	Hearing Disability Class VI	6	1.8%
Type of High School	Special Education School	282	88.1%
	General High School	30	9.3%
	General Vocational School	43	13.4%

a According to notice on the unified production and issuance of the disabled persons’ certificate of the People’s Republic of China, the classification of hearing disability is divided into four levels, so the sample population just knows their own classification criteria in China. Hearing disability class I refers to dBspL > 90 and speech discrimination score < 15%; Hearing Disability Class II refers to that dBspL ranges from 71–90 and speech discrimination score ranges from 15–30; Hearing Disability Class III refers to that dBspL and speech discrimination score ranges from 61–70 and 31–60; Hearing Disability Class VI, 51–60 (dBspL), 61-70(speech discrimination score).

## Results

### Descriptive statistics

In the descriptive analysis table, the age distribution of participants was mainly 21 (27.8%) and 22 (20.9%), followed by 20 (19.6%) and 19 (11.2%), indicating that the age composition of respondents was the young group, with few respondents under 17 and over 27 or older, accounting for only 1.5% of the total. In terms of gender ratio, there were slightly more female participants (57.1%) than male (42.8%). As for registratered residence types, the proportion of rural registration and those living in rural areas accounted for the highest, reaching 61.8%, while only 14.3% of urban registration lived in urban areas, that is to say that most of the respondents were from rural backgrounds. The proportion of participants with hearing disability level I reached 73.1%, and level II is 12.8%, which means the majority of participants suffers severe hearing disablity. Besides, the vast majority of respondents (88.1%) came from special education schools, while the participants in regular senior middle schools and regular vocational schools were relatively small, accounting for 9.3 and 13.4%, respectively. It reflects that the respondents in this survey are mainly young and disabled from rural areas, and most of them receive education in special education schools (see Table 2).

**Table 2:** Frequency of social media use analysis.

Social media	Frequency	Percentage
WeChat	310	93.9
Douyin (Tiktok)	230	71.9
Kuaishou	219	68.4
Bilibili	71	22.2
Weibo	123	38.4
QQ	173	54.1
XiaoHongShu (Red)	69	21.6
Tieba (online forum)	22	6.9
Douban	15	4.7
Momo, Soul and other dating apps	4	1.3
Other	13	4.1

In the above social media, Wechat and QQ are communication platforms among closer friends, Douyin, kuaishou, bilibili are video platforms, and Weibo, Xiaohongshu, Tieba and Douban are communication platforms among users who are distant from each other. WeChat ranks first with a 93.9% usage rate, while TikTok and Kuaishou follow with 71.9 and 68.4%, respectively. The short videos and live broadcast functions of the latter two platforms attracted a large number of D/HH college students, and visual content can be enjoyed without relying on hearing. The usage rate of Bilibili, Xiaohongshu (Red) is relatively low, with 22.2 and 21.6%, respectively. Tieba, Douban and other platforms’ use rate is particularly low, with all of them below 7%. By analyzing the software used by the hearing impaired, it can be concluded that the hearing impaired are more inclined to use comprehensive, easy to operate and rich content social software.

### Answering research question

RQ1 asked what are the major reasons for social media use among Chinese D/HH college students. The reasons are varied. The primary reasons include communication, relaxation, expressing opinions, and acquiring information and knowledge. “Communication” ranks highest with an average score of 3.878, indicating that social media provides an important platform for D/HH college students to communicate, compensating for their communication barriers in real life. “Relaxation” and “expressing opinions” have average scores of 3.838 and 3.731 respectively, showing that social media plays a significant role in helping D/HH college students relax and express themselves. These data reflect that social media is not only a tool for them but also an extension of their lifestyle. “Acquiring information and knowledge” has an average score of 3.641, ranking fourth. This indicates that they also value using social media to obtain external information and broaden their knowledge. Overall, the reasons why D/HH college students use social media exhibit characteristics of diversity and practicality (see Table 3).

**Table 3:** Reasons for social media use.

	Minimum	Maximum	Mean	Standard deviation	Rank
Interpersonal communication	1	5	3.563	1.107	6
Acquiring information and knowledge	1	5	3.641	1.144	4
Passing time	1	5	3.325	1.077	8
Entertainment	1	5	3.338	1.166	7
Relaxation	1	5	3.838	1.044	2
Expressing opinion	1	5	3.731	1.028	3
Communication	1	5	3.878	1.042	1
Convenient	1	5	3.613	1.083	5

### Correlation analysis

Analysis through Table 4 reveals the correlations among the frequency of social media use, self-esteem, perceived social support, and life satisfaction. The correlation between life satisfaction and the frequency of social media use is as high as 0.597 (*p* < 0.001), demonstrating that higher life satisfaction is associated with more frequent social media use, which is consistent with the results of previous studies (Alessandra Gaia et al., 2020; Paglieri et al., 2023). Furthermore, the results also show how social media use frequency impacted other variables. The correlation coefficient between perceived social support and the frequency of social media use is 0.811 (*p* < 0.001), reaching a significant level, with a very large and strong correlation between perceived social support and social media use frequency. And the correlation coefficient between life satisfaction and perceived social support is 0.589 (p < 0.001), which refers to fact that D/HH college students with higher perceived social support have higher life satisfaction. Besides, this study finds that the correlation coefficient between self-esteem and life satisfaction is relatively high, reaching up to 0.664 (*p* < 0.001). There is also a positive correlation between self-esteem and perceived social support, demonstrating that D/HH individuals with higher levels of social support also have higher self-esteem. Overall, the results are consistent with Hypothesis 1, proving the positive correlation between social media use and life satisfaction. According to the correlation analyses, the study further investigates what the specific impact paths between several variables are.

**Table 4:** Correlation analysis of social media use frequency for D/HH.

		Self-esteem	Life satisfaction	Perceived social support	Social media use frequency
Self-esteem	Correlation coefficient	1.000	0.664**	0.557**	0.618**
	Significance (two-tailed)		<0.001	<0.001	<0.001
Life satisfaction	Correlation coefficient		1.000	0.589**	0.597*
	Significance (two-tailed)			<0.001	<0.001
Perceived social support	Correlation coefficient			1.000	0.811**
	Significance (two-tailed)				<0.001
Social media use frequency	Correlation coefficient				1.000
	Significance (two-tailed)				

*. in 0.05 level (two-tailed) represents significant correlation. ** in 0.01 level (two-talied) represents significant correlation.

### Mediation effect analysis

The current study investigates the mechanism that how the frequency of social media use affects life satisfaction through mediation effect. The direct effect shows that the effect value of social media use frequency on life satisfaction is 0.231, with a t-value of 1.964 and a significance level of 0.049, indicating that the frequency of social media use has a direct positive impact on life satisfaction, although the effect value is small, it is still statistically significant (see Table 5).

**Table 5:** Mediate effect table.

Effect	Item	Effect size	Standard error	t	P	Lower bound of 95% confidence interval	Upper limit of 95% confidence interval
Direct effect	Social media use frequency= > life satisfaction	0.231**	0.118	1.964	0.049	0	0.463
Indirect effect	Social media use frequency= > perceived social support	1.499***	0.053	28.26	<0.001	1.395	1.603
	Social media use frequency= > self-esteem	0.327***	0.077	4.246	<0.001	0.175	0.478
	Perceived social support= > self-esteem	0.170***	0.043	3.913	<0.001	0.084	0.255
	Perceived social support= > life satisfaction	0.206***	0.066	3.118	0.002	0.076	0.336
	Self-esteem= > life satisfaction	0.940***	0.084	11.247	<0.001	0.776	1.104
Total effect	Social media use frequency= > life satisfaction	1.087***	0.075	14.558	<0.001	0.94	1.234

The indirect effect of social media use frequency on perceived social support is 1.499, with a t-value of 28.26 and a significance level less than 0.001, suggesting that social media use frequency significantly enhances perceived social support. Furthermore, the effect value of social media use frequency on self-esteem is 0.327, with a t-value of 4.246 and a significance level also less than 0.001, which refers to the fact that social media use frequency significantly improves self-esteem. These results reveal that perceived social support and self-esteem play significant mediating roles on the relationship between social media use frequency and life satisfaction, which are consistent with Hypothesis 2.

Additionally, perceived social support on life satisfaction is 0.206, with a t-value of 3.118, and the effect value of perceived social support on self-esteem is 0.17, with a t-value of 3.913 and a significance level as little as 0.001. These values mean that D/HH individuals who received perceived social support from frequent social media use tend to possess higher level of self-esteem, which in turn leads to the feeling of being more satisfied with their lives. The indirect effect paths divide into two channels, one of them is “social media use frequency→perceived social support→life satisfaction,” the other is “social media use frequency→perceived social support→self-esteem→life satisfaction”.

**Figure 1:**
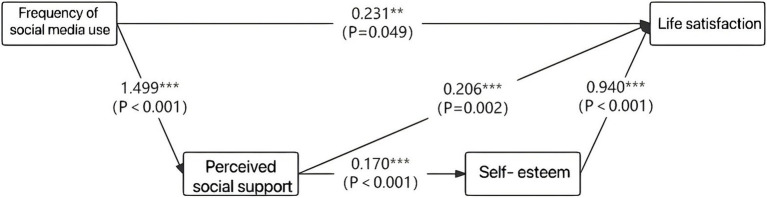
Mediating effect.

The specific statistics can be seen in Figure 1. The total effect value is 1.087, with a t-value of 14.558 and a significance level less than 0.001, demonstrating that social media use frequency has a significant comprehensive impact on life satisfaction through both direct and indirect pathways.

### Multiple linear regression

In order to confirm the reliability and completeness of this study, multiple linear regression was applied. Table 6 explains social media frequency. The R-value is 0.632, indicating that the presentation model is able to explain 63.2% of the variation in the dependent variable. The R^2^ value is 0.400, and the adjusted R^2^ value is 0.398, reflecting relatively high explanatory power of the model. The standard error of the estimate is 0.8744, indicating some prediction errors in the model. The change statistic is 0.400, showing an improvement in the model’s goodness of fit. The Durbin-Watson value is 2.032, indicating no autocorrelation of residuals and that the model fits well. The F change value is 211.948, with a corresponding significance of F change being less than 0.001, demonstrating that the model is overall significant, with the significance level of the F change being less than 0.05 (see Table 7).

**Table 6:** Regression model summary.

R	R^2^	Adjusted R^2^	Standard estimation fault	Changed values					Durbin-Watson
				R^2^ variation	F variation	Df 1	Df 2	Significance F variation	
0.632	0.400	0.398	0.8744	0.400	211.948	1	318	<0.001	2.032

**Table 7:** ANOVA.

Model	Quadratic sum	Degrees of freedom	MS	F	Significance
Regression	162.048	1	162.048	211.948	<0.001
Residual error	243.131	318	0.765		
Total	405.179	319			

According to the analysis based on the ANOVA table, the sum of squares for the regression model is 162.048, with df 1, mean square 162.048, *F*-value of 211.948, and a significance level less than 0.001. This indicates that the regression model has a very strong explanatory power for the data and the results are highly statistically significant. The sum of squares for the residuals is 243.131, with df 318, and MS 0.765, indicating that the unexplained variance by the model is relatively small. Overall, the model’s F-value is high, and the significance level is extremely low, suggesting that the independent variables have a significant impact on the dependent variable, and the model’s fit is favorable. Therefore, the established regression model can be considered effective. The total sum of squares is 405.179, with degrees of freedom of 319, confirming the overall explanatory power of the model (see Table 8).

**Table 8:** Regression coefficient table.

	Unstandardized coefficients		Standardized coefficients	t	significance	Collinearity statistics	
	B	Standard error	Beta			Tolerance	VIF
(Constant)	1.396	0.174		8.032	<0.001		
Social media use frequency	1.087	0.075	0.632	14.558	<0.001	1.000	1.000

In the regression coefficients table, the coefficient for the constant term is 1.396, with a standard error of 0.174, and a corresponding t-value of 8.032 at a significance level of <0.001, indicating that the constant term is highly significant statistically. Among the single independent variable, the unstandardized coefficient for social media use frequency is 1.087, with a standard error of 0.075, a standardized coefficient of 0.632, a t-value of 14.558, and a significance level of <0.001, demonstrating that the frequency of social software usage has a significant positive impact on the dependent variable which is life satisfaction. The tolerance and VIF values in the collinearity statistics are both 1.000, indicating no collinearity issues between social software usage frequency and the constant term (see Table 9).

**Table 9:** Residual statistics table.

	Minimum	Maximum	Mean	Standard deviation
Predicted value	2.483	6.830	3.824	0.713
Residual error	−3.242	3.028	0.000	0.873
Normal expected value	−1.882	4.217	0.000	1.000
Standard residuals	−3.707	3.463	0.000	0.998

According to the table above, the distribution of model predicted values and residuals can be observed. The minimum predicted value is 2.483, the maximum is 6.830, the mean is 3.824, and the standard deviation is 0.713, demonstrating a relatively concentrated distribution of predicted values in the model. The minimum residual is −3.242, the maximum is 3.028, and the mean is 0.000, showing that the residuals are well-centered overall. The standard deviation of the residuals is 0.873, indicating a relatively dispersed distribution of residuals, implying some prediction errors in the model. The ranges of standardized predicted values and standardized residuals are from −1.882 to 4.217 and from −3.707 to 3.463, respectively, with both having a mean of 0.000 and standard deviations of 1.000 and 0.998, respectively, indicating a reasonable distribution of standardized data. Overall, the distribution characteristics and standard deviation of the residuals suggest that the model predictions are fairly accurate, and the fluctuation of residuals is within an acceptable range, further validating the stability and reliability of the model.

## Discussion

The results of the current study reveal a significant positive correlation between the frequency of social media use and life satisfaction for D/HH. In this association, the mediating effects of perceived social support and self-esteem play a crucial role. This paper not only meticulously examines the mechanism of these mediating factors and elaborates on how these variables bridge the gap between social media usage frequency and life satisfaction but also specifically focuses on China, a country with the largest population of individuals with hearing impairments, providing valuable insights into the social media behaviors of the D/HH community within a specific cultural context. In line with several new studies, the current research further encourages us to consider how to enhance social participation among minority groups (Escobar-Viera et al., 2020; Hwang and Kim, 2015), represented by the D/HH population, in the rapidly evolving landscape of social media, and how this emerging platform multidimensionally impacts their mental health. Additionally, this research employs a quantitative thematic analysis method to precisely identify the four main reasons why D/HH college students use social media: communication, expressing personal views, obtaining information and knowledge, and relaxation and entertainment. Among these, communication emerges as the most common reason for using social media, reinforcing the unique value of social media in facilitating social interactions for the D/HH community. This finding aligns with previous research (Antonucci et al., 2017; Whiting and Williams, 2013), which collectively emphasizes the importance of information seeking and communication in social media use. D/HH individuals can fully leverage written, visual, and multi-interfaces communication features offered by social media (O’Brien and Kusters, 2017; Chang, 2014), which provides them with numerous advantages and new opportunities of communicating. Through social media, D/HH individuals can engage in equal and barrier-free communication with hearing individuals without the concern of exposing their impairments due to hearing loss (Alper and Goggin, 2017), allowing them to express their opinions more confidently, participate in interpersonal interactions, and establish closer social connections. The following sections will explore the mediating mechanisms of media accessibility, perceived social support, and self-esteem, as well as their implications for the mental health of D/HH individuals.

Firstly, there is a significant characteristic in its sample selection, with respondents studying in special education schools comprising as much as 88.1% of all participants, significantly exceeding the proportion of general school or technical school, making up only 11.9%. This finding reveals that the majority of respondents study in special education schools, which is a key indicator that has not been explicitly mentioned or thoroughly explored in previous research. Given the substantial division between D/HH community and hearing environment (Maxwell-McCaw and Zea, 2011), D/HH individuals who have been educated in special schools often face more severe challenges compared to others (Johansson et al., 2021). They not only receive less social attention but also have relatively limited opportunities and abilities to access and utilize social media. Studies also indicates that the disabled have lower internet access rates and more difficulties to achieve online participation than the hearing people (Johansson et al., 2021; Scholz et al., 2017; Dobransky and Hargittai, 2016) and this chasm inclines to widen in the digital era (Nam and Park, 2017). Consequently, this population exhibits a generally low frequency of social media use, with an average using time of only 2.3 h a day. The positive relationship between social media use and life satisfaction among D/HH group demonstrated in the study suggests the urgency and significance to provide more social media access for people with disabilities while it is a convenient way for them to increase social participation.

Despite the remarkable speed of social media development and its increasing prevalence in recent years, special populations still struggle to effectively integrate into the hearing environment, and their ability and avenues for obtaining information remain significantly constrained. Maxwell-McCaw and Zea (2011), in his research, identifies four stages of identity recognition that D/HH individuals need to navigate, with the first stage “marginalization,” where individuals lack a clear sense of identity affiliation. Many deaf children grow up in a hearing family whose parents may not understand the deaf experience, making it difficult for them to acquire oral language, values, and behavioral norms from the auditory environment as they develop. As they becoming mature, D/HH individuals gain more life experience, yet their integration into the education sector remains concerning. From the perspective of high school education, the vast majority of the D/HH population continues to receive education in special education schools, which somewhat facilitates the development of their own groups. Although this educational environment provides a relatively concentrated learning and communication space for D/HH individuals, it limits their interaction and integration with hearing individuals, thereby increasing the challenges they face in adapting to hearing environments and achieving acculturation.

Secondly, the findings of this study reveal a notable positive correlation between the frequency of social media use and life satisfaction, with this correlation further detailed into direct and indirect effects. This observation aligns with previous research focusing on the impact of the use of social media on users’ psychosocial adaptation (Park, 2023; Paglieri et al., 2023; Alessandra Gaia et al., 2020), supporting Hypothesis 1 of this study. Specifically, social media has become an indispensable channel for D/HH groups to communicate and build social connections, obtain information and knowledge, and enhance life satisfaction (Schäfer and Miles, 2023; Rachdito and Hidayat, 2022; Williams et al., 2012; Power and Power, 2004, 2009).

On the one hand, given that D/HH individuals are especially vulnerable to feelings of isolation (Solomon, 2012), the anonymity feature of social media platforms may alleviate social anxiety induced by negative emotions such as inferiority and discomfort among them. This alleviation effect might help them align their real lives more closely with their psychological expectations, thereby improving their overall life satisfaction. In other words, the anonymity and convenience of online interactions provide a relatively stress-free social environment for D/HH individuals, allowing them to express themselves more freely, thus enhancing their life satisfaction.

On the other hand, the extensive platforms and abundant information resources offered by social media effectively break the boundaries between the traditional hearing environment and the D/HH living space, creating favorable conditions for the integration of D/HH individuals into the broader social environment, which means they can be regarded as general people (Paglieri et al., 2023). This integration not only promotes the social participation of hearing-impaired individuals but also aids in improving their mental health (Schäfer and Miles, 2023), thereby enhancing their life satisfaction.

Thirdly, consistent with Hypothesis 2, our findings clearly reveal the mediating role of perceived social support in the relationship between the frequency of social media use and life satisfaction. Specifically, we found a significant positive correlation between the frequency of social media use and individuals’ levels of social support all in line with previous research results (Paglieri et al., 2023; Caba Machado et al., 2023; Lin et al., 2022; Lee and Cho, 2019), which constitutes the first stage effect: “social media use frequency → perceived social support.” In this stage, individuals establish and maintain positive and trustworthy interpersonal networks through social media platforms, leading to a heightened perception of social support (Frison and Eggermont, 2016). When individuals form such positive relationships within their social networks, they experience effective emotional release and find solutions to the challenges they face. This positive emotional experience and problem-solving ability encourage individuals to feel happier subjectively while reducing feelings of loneliness. This transition process—“perceived social support → life satisfaction”—also exhibits a positive correlation, further validating the importance of social support in enhancing life satisfaction.

While previous studies have not explained how these two mediators come into effect concretely, it is worth noting that our data also reveal a sequential response mechanism. Social support obtained from friends, family, and significant others not only directly enhances individuals’ life satisfaction but also exerts deeper positive effects on life satisfaction by influencing self-esteem as a mediating variable. This continuity response pathway—“social media use frequency → perceived social support → self-esteem → life satisfaction”—is supported by our study. The self-esteem levels of D/HH individuals can be influenced by several factors, including poor parental communication skills, insufficient maternal bonding, feelings of mistrust stemming from perceived inequality and negative attitudes toward deaf individuals, social isolation, negative body image, lack of a robust cultural identity, and rejection by family members and society at large (Mulcahy, 1998), and through using social media, they can receive a sense of collective identity (Ellcessor, 2018; Lomicky and Hogg, 2010), as offered by online friends or important others, leading to a reduction in self-criticism and negativity while allowing them to feel valued, thus resulting in higher levels of life satisfaction. This indicates that the mediating effect of self-esteem between social media use and life satisfaction is achieved through the bridging role of social support, consistent with our Hypothesis 3. These resources and support not only directly enhance their subjective well-being but also further improve their life satisfaction by boosting self-esteem as a psychological mechanism. The indirect impact of self-esteem may be influenced by various factors (Hayes, 2018), including moderators like the personality traits (Seidman, 2013) and degree of disability (Hwang and Nam, 2021).

## Limitations and future research

This paper considers the influence of two mediators on the relationship between social media use and life satisfaction, revealing two indirect effect pathways. It also addresses the social background and media engagement capabilities of D/HH groups who are diagnosed as deaf, as well as the diverse software options available for social media. However, there are still several limitations, which can provide insights for future related research.

Firstly, the current sample group’s age, household registration types, geographical distribution and education background are not sufficiently balanced, which is also significant for the results. Despite the age range being from 17 to 42, the majority of participants are concentrated in the younger demographic of 19 to 24 years, with very few middle-aged individuals and no elderly participants. Additionally, D/HH college students are educated and have master of social media skills who possess some kind of privilege compared with other D/HH groups. And the sample of this study does not consider the situation of newborn hearing screening or hearing aids. To more accurately reflect the relationships among the various variables, future studies can expand the sample size, and balance the representation of individuals from different age groups, household registration types and literacy skills to avoid potential confounding effects.

Secondly, the current study only considers perceived social support and self-esteem as mediators and does not account for the regulatory effects of additional mediators or moderators. While perceived social support and self-esteem have well-established and reliable scales, making research on them standardized and normative, future studies could innovatively include factors such as personality traits, social capital, and degree of disability, developing new scales for more comprehensive research.

Thirdly, while the independent variable in this study is the frequency of social media use, the measurement of social media usage should not be limited to frequency alone; it also includes intensity, duration, number of friends in social circles, and account quantity. Therefore, the independent variable could be modified, or the aforementioned aspects could be integrated to form a more comprehensive independent variable.

## Data Availability

The original contributions presented in the study are included in the article/supplementary material, further inquiries can be directed to the corresponding author.

## References

[ref1] Alessandra Gaia E. Sala E. Cerati G. (2020). Social networking sites use and life satisfaction. A quantitative study on older people living in Europe. Eur. Soc. 23, 98–118. doi: 10.1080/14616696.2020.1762910

[ref2] Alper M. Goggin G. (2017). Digital technology and rights in the lives of children with disabilities. New Media Soc. 19, 726–740. doi: 10.1177/1461444816686323

[ref3] Antonucci T. C. Ajrouch K. J. Manalel J. A. (2017). Social relations and technology: Continuity, context, and change. Innov. Aging 1, 1–9. doi: 10.1093/geroni/igx029, PMID: 29795794 PMC5954608

[ref4] Blachnio A. Przepiorka A. Rudnicka P. (2016). Narcissism and self-esteem as predictors of dimensions of Facebook use. Personal. Individ. Differ. 90, 296–301. doi: 10.1016/j.paid.2015.11.018

[ref5] Bradfield O. M. (2021). Hearing parents voices: Parental refusal of cochlear implants and the zone of parental discretion. J. Bioethic. Inquiry 19, 143–150. doi: 10.1007/s11673-021-10154-8, PMID: 34918184 PMC9007755

[ref6] Caba Machado V. Mcilroy D. Padilla Adamuz F. M. Murphy R. Palmer-Conn S. (2023). The associations of use of social network sites with perceived social support and loneliness. Curr. Psychol. 42, 14414–14427. doi: 10.1007/s12144-021-02673-9, PMID: 35103040 PMC8791808

[ref7] Chang C.-M. (2014). New media, new technologies and new communication opportunities for Deaf/Hard of Hearing people. Online J. Commun. Media Technol. 4, 38–52. doi: 10.30935/ojcmt/5703

[ref8] Çivitci N. Çivitci A. (2009). Self-esteem as mediator and moderator of the relationship between loneliness and life satisfaction in adolescents. Personal. Individ. Differ. 47, 954–958. doi: 10.1016/j.paid.2009.07.022

[ref9] Cohen S. Hoberman H. M. (1983). Positive events and social supports as buffers of life change stress. J. Appl. Soc. Psychol. 13, 99–125. doi: 10.1111/jasp.1983.13.issue-2

[ref10] Coleman J. S. (1988). Social capital in the creation of human capital. Am. J. Sociol. 94, S95–S120. doi: 10.1086/228943

[ref11] Crocker J. Canevello A. (2008). Creating and undermining social support in communal relationships: The role of compassionate and self-image goals. J. Pers. Soc. Psychol. 95, 555–575. doi: 10.1037/0022-3514.95.3.555, PMID: 18729694

[ref12] Denti L. Barbopoulos I. Nilsson I. Holmberg L. Thulin M. Wendeblad M.. (2012). Sweden’s largest Facebook study, vol. 3. Goteborg: Gothenburg Research Institute.

[ref13] Diener E. Emmons R. A. Larsen R. J. Griffin S. (1985). The Satisfaction With Life Scale. J. Pers. Assess. 49, 71–75. doi: 10.1207/s15327752jpa4901_13, PMID: 16367493

[ref14] Ding Q. Zhang Y. X. Wei H. Huang F. Zhou Z. K. (2017). Passive social network site use and subjective well-being among Chinese university students: A moderated mediation model of envy and gender. Personal. Individ. Differ. 113, 142–146. doi: 10.1016/j.paid.2017.03.027

[ref15] Dobransky K. Hargittai E. (2016). Unrealized potential: Exploring the digital disability divide. Poetics 58, 18–28. doi: 10.1016/j.poetic.2016.08.003

[ref16] Eichengreen A. Almog N. Broyer N. (2016). “The disability closet: Coming out, staying in, or deconstructing” in Disability studies: A reader. eds. Mor S. Ziv N. Kanter A. Eichengreen A. Mizrachi N. (Jerusalem, Israel: Van Leer Institute), 299–311.

[ref17] Ellcessor E. (2018). One tweet to make so much noise: Connected celebrity activism in the case of Marlee Matlin. New Media Soc. 20, 255–271. doi: 10.1177/1461444816661551

[ref18] Escobar-Viera C. Shensa A. Hamm M. Melcher E. M. Rzewnicki D. I. Egan J. E.. (2020). “I don’t feel like the odd one”: Utilizing content analysis to compare the effects of social media use on well-being among sexual minority and nonminority US young adults. Am. J. Health Promot. 34, 285–293. doi: 10.1177/0890117119885517, PMID: 31698919 PMC7404611

[ref19] Frison E. Eggermont S. (2016). Exploring the relationships between different types of Facebook use, perceived online social support, and adolescents’ depressed mood. Soc. Sci. Comput. Rev. 34:153171, 153–171. doi: 10.1177/0894439314567449

[ref20] Glickman N. S. (1993). Deaf identity development: Construction and validation of a theoretical model. (Unpublished doctoral dissertation). Amherst, MA: University of Massachusetts.

[ref21] Glickman N. S. Carey J. C. (1993). Measuring deaf cultural identities: A preliminary investigation. Rehabil. Psychol. 38, 275–283. doi: 10.1037/h0080304

[ref22] Hawi N. S. Samaha M. (2017). The relations among social media addiction, self-esteem, and life satisfaction in university students. Soc. Sci. Comput. Rev. 35, 576–586. doi: 10.1177/0894439316660340

[ref23] Hayes A. F. (2018). Introduction to mediation, moderation, and conditional process analysis. 2nd Edn. New York: The Guilford Press.

[ref24] Helms J. E. (1990). Black and white racial identity: Theory, research, and practice. Westport, CT: Greenwood Press.

[ref25] Hintermair M. (2008). Self-esteem and satisfaction with life of deaf and hard of hearing people: A resource-oriented approach to identity work. J. Deaf Stud. Deaf Educ. 13, 278–300. doi: 10.1093/deafed/enm054, PMID: 17971343

[ref26] Hunsinger J. Senft T. M. (2013). The social media handbook. New York, NY: Routledge.

[ref27] Hutto C. J. Bell C. Farmer S. Fausset C. Harley L. Nguyen J.. (2015). Social media gerontology: Understanding social media usage among older adults. Web Intell. 13, 69–87. doi: 10.3233/WEB-150310

[ref28] Hwang H. Kim K.-O. (2015). Social media as a tool for social movements: The effect of social media use and social capital on intention to participate in social movements. Int. J. Consum. Stud. 39, 478–488. doi: 10.1111/ijcs.12221

[ref9001] Hwang H. Nam S. (2021). Social media use and subjective well‐being among middle‐aged consumers in Korea: Mediation model of social capital moderated by disability. J. Consum. Aff. doi: 10.1111/joca.12354

[ref29] Jan M. Soomro S. A. Ahmad N. (2017). Impact of social media on self-esteem. ESJ 13, 329–341. doi: 10.19044/esj.2017.v13n23p329

[ref30] Johansson S. Gulliksen J. Gustavsson C. (2021). Disability digital divide: The use of the internet, smartphones, computers, and tablets among people with disabilities in Sweden. Univ. Access Inf. Soc. 20, 105–120. doi: 10.1007/s10209-020-00714-x

[ref31] Kaplan A. M. Haenlein M. (2010). Users of the world, unite! The challenges and opportunities of Social Media. Bus. Horiz. 53, 59–68. doi: 10.1016/j.bushor.2009.09.003

[ref32] Kietzmann J. H. Hermkens K. McCarthy I. P. Silvestre B. S. (2011). Social media? Get serious! Understanding the functional building blocks of social media. Kelley School Business Indiana Univ. 54, 241–251. doi: 10.1016/j.bushor.2011.01.005, PMID: 39939421

[ref33] Kirkpatrick L. A. Ellis B. J. (2001). “An evolutionary-psychological approach to self-esteem: Multiple domains and multiple functions” in Blackwell handbook of social psychology: Interpersonal processes. eds. Fletcher G. J. O. Clark M. S. (Oxford, UK: Blackwell Publishers), 411–436.

[ref34] Kushalnagar P. Kushalnagar R. (2018). “Health-related information seeking among deaf adults: findings from the 2017 Health Information National Trends Survey in American Sign Language (HINTS-ASL)” in eHealth: Current Evidence, Promises, Perils and Future Directions (Studies in Media and Communications, Vol. 15). eds. Hale T. M. Chou W.-Y. S. Cotten S. R. Khilnani A. (Leeds: Emerald Publishing Limited).

[ref35] Lambez T. Nagar M. Shoshani A. Nakash O. (2020). The association between deaf identity and emotional distress among adolescents. J. Deaf. Stud. Deaf. Educ. 25, 251–260. doi: 10.1093/deafed/enz051, PMID: 32034400

[ref36] Lariscy R. W. Tinkham S. F. Sweetser K. D. (2011). Kids these days: Examining differences in political uses and gratifications, internet political participation, political information efficacy, and cynicism on the basis of age. Am. Behav. Sci. 55, 749–764. doi: 10.1177/0002764211398091

[ref37] Leary M. R. Tambor E. S. Terdal S. K. Downs D. L. (1995). Self-esteem as an interpersonal monitor: The sociometer hypothesis. J. Pers. Soc. Psychol. 68, 518–530. doi: 10.1037/0022-3514.68.3.518

[ref38] Lee H. E. Cho J. (2019). Social media use and well-being in people with physical disabilities: Influence of SNS and online community uses on social support, depression, and psychological disposition. Health Commun. 34, 1043–1052. doi: 10.1080/10410236.2018.1455138, PMID: 29652521

[ref39] Li X. Chen W. Popiel P. (2015). What happens on Facebook stays on Facebook? The implications of Facebook interaction for perceived, receiving, and giving social support. Comput. Hum. Behav. 51, 106–113. doi: 10.1016/j.chb.2015.04.066

[ref40] Lin S. Liu D. Niu G. Longobardi C. (2022). Active social network sites use and loneliness: The mediating role of social support and self-esteem. Curr. Psychol. 41, 1279–1286. doi: 10.1007/s12144-020-00658-8

[ref41] Lomicky C. S. Hogg N. M. (2010). Computer-mediated communication and protest. Inf. Commun. Soc. 13, 674–695. doi: 10.1080/13691180903214515

[ref42] Maxwell-McCaw D. Zea M. C. (2011). The Deaf Acculturation Scale (DAS): Development and validation of a 58-item measure. J. Deaf. Stud. Deaf. Educ. 16, 325–342. doi: 10.1093/deafed/enq061, PMID: 21263041 PMC4723658

[ref43] Michel J. S. Mitchelson J. K. Pichler S. Cullen K. L. (2010). Clarifying relationships among work and family social support, stressors, and work–family conflict. J. Vocat. Behav. 76, 91–104. doi: 10.1016/j.jvb.2009.05.007

[ref44] Miller R. A. (2017). “My voice is definitely strongest in online communities”: Students using social media for queer and disability identity making. J. Coll. Stud. Dev. 58, 509–525. doi: 10.1353/csd.2017.0040

[ref45] Mulcahy R. T. (1998). Cognitive self-appraisal of depression and selfconcept: Measurement alternatives for evaluating affective states. Unpublished doctoral dissertation. Washington: Gallaudet University.

[ref46] Nam S.-J. Park E.-Y. (2017). The efects of the smart environment on the information divide experienced by people with disabilities. Disabil. Health J. 10, 257–263. doi: 10.1016/j.dhjo.2016.11.001 27899268

[ref47] National Disability Survey Leadership Group, Second National Disability Survey Office. (2024). Peking University Open Research Data Platform VI. Beijing: Second National Disability Survey.

[ref48] O’Brien D. Kusters A. (2017). Visual methods in deaf studies: Using photography and filmmaking in research with deaf people. In Kusters A. Meulder M. De O’Brien D. (Eds.), Innovations in deaf studies: the role of deaf scholars (pp. 265–296). Oxford: Oxford University Press.

[ref49] Oh H. J. Ozkaya E. LaRose R. (2014). How does online social networking enhance life satisfaction? The relationships among online supportive interaction, affect, perceived social support, sense of community, and life satisfaction. Comput. Hum. Behav. 30, 69–78. doi: 10.1016/j.chb.2013.07.053

[ref50] Oshio T. Kimura H. Nishizaki T. Omori T. (2020). Association between the use of social networking sites, perceived social support, and life satisfaction: Evidence from a population-based survey in Japan. PLoS One 15:e0244199. doi: 10.1371/journal.pone.0244199, PMID: 33338072 PMC7748275

[ref51] Paglieri T. A. Schooler D. Pezzarossi C. K. (2023). Social networking site usage of middle-aged and older deaf adults. J. Deaf. Stud. Deaf. Educ. 28, 311–326. doi: 10.1093/deafed/enad003, PMID: 36906844

[ref52] Park E.-Y. (2023). The relation among smartphone use, social capital, and life satisfaction in individuals with physical disabilities. Curr. Psychol. 43, 11538–11545. doi: 10.1007/s12144-023-05275-9, PMID: 39944678

[ref53] Pavot W. Diener E. (1993). Review of the Satisfaction With Life Scale. Psychol. Assess. 5, 164–172. doi: 10.1037/1040-3590.5.2.164

[ref54] Pavot W. Diener E. (2008). The Satisfaction With Life Scale and the emerging construct of life satisfaction. J. Posit. Psychol. 3, 137–152. doi: 10.1080/17439760701756946

[ref55] Power M. R. Power D. (2004). Everyone here speaks TXT: deaf people using SMS in Australia and the rest of the world. J. Deaf. Stud. Deaf. Educ. 9, 333–343. doi: 10.1093/deafed/enh042, PMID: 15304436

[ref9002] Power D. Power M. R. (2009). Communication and culture: signing deaf people in Europe. Technol. Disabil. 21, 127–134. doi: 10.3233/TAD-2009-0287

[ref56] Rachdito E. B. Hidayat Z. (2022). Emoticons as self disclosure in social media and its meaning for people who are deaf. Disabil. CBR Inclus. Dev. 32, 40–62. doi: 10.47985/dcidj.471

[ref57] Rosenberg M. (1965). Society and the adolescent self-image. Princeton: Princeton University Press.

[ref58] Schäfer K. Miles F. (2023). Social media use and mental health in deaf or hard-of-hearing adults—Results of an online survey. Front. Commun. 8:1175461. doi: 10.3389/fcomm.2023.1175461

[ref59] Scholz F. Yalcin B. Priestley M. (2017). Internet access for disabled people: Understanding socio-relational factors in Europe. Cyberpsychology 11:4. doi: 10.5817/CP2017-1-4

[ref60] Seidman G. (2013). Self-presentation and belonging on Facebook: How personality influences social media use and motivations. Personal. Individ. Differ. 54, 402–407. doi: 10.1016/j.paid.2012.10.009

[ref61] Sherbourne C. D. Stewart A. L. (1991). The MOS social support survey. Soc. Sci. Med. 32, 705–714. doi: 10.1016/0277-9536(91)90150-B, PMID: 2035047

[ref62] Shin D. C. Johnson D. M. (1978). Avowed Ed Diener happiness as an overall assessment of the quality of life. Soc. Indic. Res. 5, 475–492. doi: 10.1007/BF00352944

[ref63] Smith E. R. Mackie D. M. Claypool H. M. (2014). Social psychology. New York, NY: Psychology Press.

[ref64] Solomon A. (2012). Far from the tree: parents, children and the search for identity. New York, NY: Scribner.

[ref65] Sparrow R. (2005). Defending deaf culture: The case of cochlear implants. J. Polit. Philos. 13, 135–152. doi: 10.1111/j.1467-9760.2005.00217.x

[ref66] Statista. (2024). Available at: https://www.statista.com/topics/1164/social-networks/#topicOverview (Accessed November 17, 2024)

[ref67] Trepte S. Dienlin T. Reinecke L. (2015). Influence of social support received in online and offline contexts on satisfaction with social support and satisfaction with life: A longitudinal study. Media Psychol. 18, 74–105. doi: 10.1080/15213269.2013.838904

[ref68] Valenzuela S. Park N. Kee K. F. (2009). Is there social capital in a social network site? Facebook use and college students’ life satisfaction, trust, and participation. J. Comput.-Mediat. Commun. 14, 875–901. doi: 10.1111/j.1083-6101.2009.01474.x

[ref69] Whiting A. Williams D. (2013). Why people use social media: A uses and gratifications approach. Qual. Mark. Res. Int. J. 16, 362–369. doi: 10.1108/QMR-06-2013-0041

[ref70] Williams D. L. Crittenden V. L. Keo T. McCarty P. (2012). The use of social media: an exploratory study of uses among digital natives. J. Public Aff. 12, 127–136. doi: 10.1002/pa.1414

[ref71] Wilson J. F. Embree J. Guthmann D. Sligar S. R. Titus J. C. (2020). Satisfaction with life scale in American Sign Language: Validation and normative data. JADARA 53, 1–10.

[ref72] World Health Organization. (2021). World report on hearing (ISBN: 978–92-4-002048-1). Available at: https://www.who.int/publications/i/item/9789240020481 (Accessed December 10, 2024).

[ref73] Yang S. Huang L. Zhang Y. Zhang P. Zhao Y. C. (2021). Unraveling the links between active and passive social media usage and seniors’ loneliness: A field study in aging care communities. Internet Res. 31, 2167–2189. doi: 10.1108/INTR-08-2020-0435

[ref74] Zimet G. D. Dahlem N. W. Zimet S. G. Farley G. K. (1988). The multidimensional scale of perceived social support. J. Pers. Assess. 52, 30–41. doi: 10.1207/s15327752jpa5201_2 2280326

